# Scrofula

**Published:** 1845-01

**Authors:** 


I. RECHERCHES ET OBSERVATIONS SUR LES CAUSES DES MALADIES SCROFULEUSES. Par *J. G. A. Lugol*, Medecin de l'Hôpital St. Louis, &c. &c. Octavo, pp. 372. Paris, 1844. Fortin et Masson.

II. RESEARCHES AND OBSERVATIONS ON THE CAUSES OF SCROFULOUS DISEASES. By *J. G. Lugol.* Translated from the French, with an Introduction, and an Essay on the Treatment of the principal Varieties of Scrofula, by *W. Harcourt Ranking*, M.D., Physician to the Suffolk General Hospital. Octavo, pp. 306. London, 1844. Churchill.

III. SCROFULA; ITS NATURE, CAUSES, AND TREATMENT, AND ON THE PREVENTION AND ERADICATION OF THE STRUMOUS DIATHESIS. By *W. Tyler Smith*, M.B. Octavo, pp. 172. London, 1844. Churchill.

THE grand aim and leading object of this new work of M. Lugol are to prove that Scrofula is a disease essentially hereditary in its origin; and that, unless there be a congenital tendency to it in the system, external causes will very rarely, if ever, induce its development in an individual; although they may not unfrequently do so in his offspring. The motto affixed to the title-page—*la santé des enfans tire son origine de la santé des parents*—may be considered as the text on which the author discourses with great volubility and earnestness through upwards of 300 pages. Like all very ardent enquirers, he may be inclined to push particular views a little too far; but, in the main, he is unquestionably right; and his elaborate and extensive researches are calculated, we think, to elucidate a very important point of pathological research.

Scrofulous disease manifests its baneful effects from the very earliest months of intra-uterine life. A vast number of cases of Abortion are attributable to its influence on the system, either of the mother or of her offspring. After birth, it arrests the moral, as well as the physical, development of the child, impressing its stamp upon every disease to which it is subject, and vitiating more or less profoundly all the successive evolutions of infancy and youth. At a more advanced period of life, it reveals its existence by a great multitude of morbid conditions and phenomena—the common origin of which has been too generally overlooked by medical men, and which, accordingly, some of our most-esteemed authors have very erroneously described as particular and individual maladies.

No part of the body is exempt from the invasion of scrofulous disease; although certain parts or tissues usually suffer much more than others. In not a few cases, the mucous membranes are chiefly affected: hence the Ophthalmias, Coryzas, Leucorrhœas, mucous Fevers, intestinal Worms, &c., that are so frequently met with in practice. If the skin be the principal seat of the disease, we find that there is an unusual liability to Chilblains, various sorts of Eczematous and Impetiginous eruptions, and obstinate ulcerations; also to *pedicular* maladies, "which,"—says M. Lugol—"like the verminous affections of the bowels, continue to be re-produced until the constitution of the patient be regenerated."

When the cellular tissue is most affected, then we have the formation of abscesses in various parts; when the bones are so, Caries, Necrosis, and Rachitis are most frequently the consequences.

Now, however much these various maladies may differ from each other in their outward appearances, we should bear in mind that they are all the offspring of one common morbid state,—the difference being only in their *seat*, or, in other words, in the tissue affected. Hence it is that we often see more than one form of the disease developed simultaneously in the same individual; while, in other patients, there is a renewed succession of different forms, following and perhaps alternating with each other.

Properly speaking, says our author, there is no Scrofula of any single tissue or organ. In all cases, it is the same disease, which in one patient indeed may affect the skin or mucous membranes, and in another the bones, &c.—but without ever fixing itself upon any of them in an isolated manner. The invasion of any one organ is sufficient in itself to make us dread the development of the evil in some other part of the body. In the same manner, as we shall afterwards find, whenever the disease manifests itself in one member of a family, there is, in that circumstance alone, a strong presumption that it will appear in some, if not in all, of the other children.

If it be asked, What is Scrofula, and wherein does it consist?—we must frankly acknowledge that we cannot answer the question when so put. All that we are prepared to affirm is, that this *vice* or morbid state is congenital, and is always manifested by the development of tubercles. This production or deposit is in truth the disease itself, its anatomical and pathognomonic sign, that which characterises it, and which gives value to all the other symptoms. In other words, whenever a patient is affected with tubercles—no matter where they are situated—he is, in our opinion, scrofulous. Whether he is affected with an ophthalmia, a cold abscess, a caries of the bones, a white-swelling, &c., the nature of these diseases cannot, and ought not to, be questioned: they are all scrofulous, if there have been tubercles, or if there is a coincidence of these morbid productions, either in the patient himself, or in some member of his family.

A Tubercle may be regarded, says M. Lugol, as having the same origin and mode of formation as any of the ordinary organs of an animal body. It is itself a sort of organ, possessed of its peculiar life or mode of existence, just as the Liver or Spleen have theirs; like them, it undergoes a spontaneous evolution. It is a morbid production that sensibly modifies all the organic elements, and deeply affects their functions; and which stamps every person, in whom it exists, with a peculiar complexion, and physiognomy—characteristic of the tuberculous disposition. It is with this peculiar complexion or constitution that the immense number of diseases, improperly termed scrofulous, is associated.

The particular nature and seat of Scrofulous affections vary a good deal, according to the age of the individual: the same person is differently affected at different periods of life. In infancy and childhood, the most common forms are chilblains, intestinal worms, the production of lice, ophthalmia, and bronchitis. At, and about, the period of puberty, the signs of tuberculous disease of the lungs—with or without the preceding maladies—usually make their appearance; and at a more advanced age, the formation of cold abscesses, white-swellings, &c., are the usual manifestations of the existing Cachexy. In short, the medical history of a single scrofulous patient will often suffice to exhibit a complete nosological tableau of the disease.

As to the primary causes of Scrofula, it would seem that whatever has the effect of enfeebling the energy of the solids, and depraving the quality of the fluids, is liable to induce its development—if not immediately, at least in the next generation. Scrofulous maladies are essentially the diseases of civilization, or, more properly speaking, of the artificial mode of life that usually accompanies it.

> "They are extremely rare," (we quote from Dr. Smith) "among savage races, except when these are brought into contact with the vices of civilized life, or transplanted into an ungenial climate. With all civilized nations, on the contrary, they are well known to prevail to an immense extent, and in none perhaps more than our own. Tuberculous disorders affect, too, all the domestic animals which man has reclaimed from the savage state, and they are common among the species brought from tropical climates to our own country. Climate appears, in the first instance, to have given rise to the peculiarity of constitution most favourable to scrofula; and the wants and vices incident to civilized life seem to be the agents which have produced the disease in the soil already fitted for its reception." 3.

In a subsequent part of his work, the same writer very justly remarks:

> "The lower order of townsfolk, and indeed the upper also, though from other causes, are continually falling into a state of disorder, termed by Dr. James Johnson the *wear and tear* malady, out of which scrofula is prone to take its rise in predisposed constitutions. Large numbers live in the great towns, in cellars and underground. They present a blanched and etiolated appearance. As a class, such people are very poor, and live badly; so that here we have large masses subjected to precisely the same physical conditions,—living on scanty and unwholesome food, and deprived of light,—as those by which pathologists have been able to produce tubercular disease at will, and to any extent, in the lower animals." 37.

In a vast number of cases, we cannot trace the development of scrofulous disease to any distinct cause, save and except that of Hereditary transmission. Whatever, indeed, as we have already said, enfeebles the powers of the system, seems to promote and accelerate its formation; but it is very doubtful whether any external cause is sufficient in itself to induce its occurrence, if there be no family predisposition to the diathesis in some one form or another. Hitherto medical men have not examined with due attention the laws of hereditary transmission in reference to this too-frequent disease; they have generally confined themselves merely to the ascertaining of the bare fact, whether one of the parents of any patient may have exhibited decided marks of the Scrofulous cachexy, or not. The question we shall find, as we proceed, to be one of much more extensive application, and one which requires a much greater extent of research than has usually been given to it.

M. Lugol, on more than one occasion, alludes to the not unfrequent *seeming* exemption of one or two members of a scrofulous family from the hereditary taint, and shews how very generally, even in them, the inborn evil makes its appearance at some time of life or another. The family resemblance, which may hitherto have never been perceptible, at length—perhaps upon some accidental occurrence—becomes too obvious not to be at once recognised. Our author quotes the following case in illustration. A lady, having all the appearance of sound and healthy constitution, was the mother of several children who were decidedly scrofulous. She was much surprised at this, although one of her own sisters was in the very same condition, and another had been affected with rickets. No person could have perceived any resemblance between her and her two sisters, until they had reached *l'age de retour*. "The first time that I remarked it," says our author, "was on seeing them all in tears, upon the death of one of the children. They wept in the same manner; they had exactly the same tone of voice, the same accentation of their words; there was in all of them the same peculiar movement of the commissures of the lips, arising in part from the loss of the very same teeth; their features also were drawn down in the same manner; and altogether the external signs of relationship were most apparent."

We cannot wonder at this; all the children of a family *must* necessarily partake of essentially the same sort of constitution, however much the outward features of this may vary, in consequence of a variety of modifying circumstances on the part, alike of the parents and of their offspring. M. Lugol strenuously insists upon the truth of this position, and adduces a vast number of observations in support of it. It is the pervading principle of his whole work.

Whenever, says he, one member of a family is affected with distinct scrofulous disease, we may be sure that traces of the same malady, or at least of the diathesis which has given rise to it, will be found in the other members. There is a something in the complexion of them all that indicates a similarity of constitution. How often do we see, in the case of scrofulous children, that the skin is soft, flabby, and white; that the head is disproportionately large; that its openings are unusually wide; that the trunk and the extremities are unequally developed—the former being large and tumid, and the latter being comparatively small, the joints only excepted! In not a few instances, we observe that the median line is not in the middle of the trunk. The result of this deformity is, there seems as if there had been a faulty junction of the two lateral halves of the body; one being placed somewhat higher and more anterior than the other. This formation of the frame, arising from an inequality of evolution, M. Lugol regards as a sufficient ground for an unfavourable prognosis in diseases in general. Besides the corporeal defect now mentioned, we may state that most cases of congenital malformations—such as hare-lip, pigeon-chest, fissure of the *linea alba,* non-descent of the testicle, &c.—occur in scrofulous children.

Scrofulous persons are generally of small stature; occasionally, however, they are of unusual and even inordinate size. Both extremes may be attributed to the circumstance, that the vital powers have been deficient in that degree of energy that is required to regulate the development of the organs: hence it is that either a partial arrest, or a hypertrophic enlargement, of the body, is apt to follow.

According to M. Lugol's observation, the majority of scrofulous patients (in France) have dark or even black hair: the proportion of those with light hair is but small. A similar remark applies to the colour of the eyes and the hue of the skin: the latter is much more frequently darkish than fair. He acknowledges that his observations on these particulars are at variance with the statements of many writers; but he appeals to the wards of St. Louis Hospital in confirmation of them. The evolution of the teeth, especially those of the second dentition, is almost always tardy in such habits: and the teeth themselves are weak, friable, and apt to be discoloured. The spongy ends of the bones are large, compared with their shafts or compact centres, and also with the adjacent soft tissues.

The evolution of the generative organs is usually more tardy in scrofulous than in other children. In scrofulous youths of 16 or 17 years of age, it is not at all uncommon to find that one of the testicles has not descended into the scrotum. In girls, the catamenial function is often very irregular in its establishment; the secretion being generally either scanty or excessive, and usually accompanied with much suffering and general disturbance. The regular development of the Mammæ and Ovaria is apt to be tardy in such cases. Considering these things, we must at once perceive the importance of scrofulous youths of both sexes avoiding the premature or inordinate indulgence in sexual pleasures. One of the most certain means of fortifying their health consists in keeping all venereal passions in check, until the body has acquired its full and mature development.

The functions of the digestive organs, too, are very generally irregular and deranged in some way or another. Very often there is a deficient appetite, owing doubtless to an atony or *inertia* of the stomach—which, our author considers to be dependent upon "un etat catarrhal de meme nature que l'ophthalmie, le coryza, l'otite, les bronchites, les leucorrhées, &c." Occasionally, however, the appetite is voracious; but, even then, the food does not afford the nourishment to the system that might be expected. The alvine evacuations are seldom quite healthy. In many cases there seems to be a sort of *intestinal leucorrhœa,* giving rise to an obstinately relaxed state of the bowels: portions of the food are then very usually voided, unchanged, along with the fæces.

The skin is often very dry and liable to lichenous and impetiginous eruptions. There is a general defect of transpiration, except in the feet, hands, and arm-pits: the sweat from these parts has not unfrequently an acid and offensive smell.

Scrofulous children are often observed to be unusually apathetic, and unwilling to exert either mind or body. On too many occasions, this inaction is attributed by parents, and even medical men, to a wrong cause. The child is considered wilful and obstinate; and its want of energy is ascribed to indolence, whereas it is the result of *malaise*, if not of actual disease.

"How often," says M. Lugol very justly, "do mothers complain that their children—the daughters especially—carry themselves badly; that they let their head fall forwards, as if they could not keep it up; and that they are always lounging about or sitting down, and seem to be indifferent to what is going on about them. The girls cannot help it. They hold themselves up badly because they are not strong, because the spinal muscles are more or less atrophied; and because the muscular fibre, in general, is deficient in the requisite tonicity. To these causes we may add the defect of harmony in the different pieces of the skeleton: this is usually only the rudimentary condition of far more conspicuous deformities, that are apt to occur at the approach of puberty—sometimes before this period, rarely after it."

That our author, indeed, is apt to carry some of his notions a little too far will appear, we should think, from the following extract.

"In general," says he, "scrofulous persons cannot bear well either bodily fatigue or mental disquietude. Sometimes indeed they possess no ordinary powers of intellect; but even then there is seldom any application or sequence in their ideas. They experience no sustained vivacity, either of their physical appetites, intellectual faculties, or moral feelings; there is nothing normal, nothing strong, nothing durable about them. All the phases of their existence abort; they have neither puberty nor mature virility; their corporeal and mental development remains incomplete."

There is a good deal of vague assertion here, that is any thing but confirmed by experience. Never was there a more scrofulous habit than that of Samuel Johnson; and who will presume to say that his mind was incompletely developed?

M. Lugol devotes a separate chapter of his work to prove the close alliance, nay, the absolute identity, of Scrofulous and Tubercular diseases. Too many medical men are still inclined to regard phthisis as a mere local malady of the chest at first, and therefore to direct their treatment rather to the relief of the pulmonic symptoms than of the constitutional diathesis. *Sydenham* very emphatically announced his view of the question, when he designated phthisis by the name of "Scrofula of the Lungs"—the expression of a doctrine, says our author, of which I have acquired the anatomical proof, as I shall speedily make known in another work on Tubercles, which is already in the press. *Portal*, also, was decidedly of opinion that phthisis was truly a scrofulous disease. *Bayle* and *Laennec's* sentiments on this subject are certainly not so near the truth as those of their predecessors. The following remarks by our author deserve notice.

"The resemblance between Scrofula and Tuberculous disease is too constant and too obvious to have completely escaped the observation of these distinguished writers; but it is to be regretted that, on this point of doctrine, they should have been less enlightened and advanced in their views than *Portal.* Their silence as to the scrofulous nature of pulmonary tubercles has been very unfortunate, seeing that it has tended to foster inaccurate ideas of localisation, and thereby to encourage a system of treatment, the most dangerous that can be employed in cases of pulmonary tubercles. If these writers, who deservedly enjoy such high authority, had laboured to unmask more completely the scrofulous character of tubercles, and had they pointed out how the course of the morbid action is always accelerated by the adoption of the antiphlogistic regimen—a practice that is based upon and derived from the *alleged* inflammatory origin of tubercles—they would have contributed not a little to prevent the abuse that has been made of blood-letting in a disease which does not, generally speaking, require any depletory measures."

In a vast number of scrofulous patients, who arrive at the age of puberty, phthisical symptoms supervene sooner or later after that period of life. How often have we occasion to observe some form or another of Scrofula, such as Ophthalmia, Ozœna, intestinal Worms, Hydrocephalus, Tabes, &c. in the offspring of phthisical persons! Most scrofulous patients are ultimately carried off by tubercular disease of the lungs: and even, when they die of other diseases or from accidents, their lungs are generally found to be more or less tuberculated. We need not remind our readers that there are various stages, as well as degrees, of tubercular deposit,—from a few dispersed miliary points of a grey-coloured gelatinous substance, to the complete infiltration of one or more lobes of the lungs with the semi-organised matter.

So generally, continues M. Lugol, has Scrofula a tuberculous origin that, in two wards containing in all 84 beds, I ascertained, beyond doubt, that pulmonary Phthisis had existed in more than one half of the parents of my patients. And, indeed, even this ratio is considerably below the mark: the proportion of cases, where the hereditary origin of Scrofula is tubercular, being unquestionably higher. We may therefore with confidence assert—and the assertion is one that has many practical bearings—that almost all the children of those, who die of tubercular Phthisis, will sooner or later exhibit marks of scrofulous disease, if not of the same pulmonary disease, which proved fatal to their parents. Ought not this intimate affinity between Scrofulous and genuinely Tuberculous disease to lead medical men to adopt similar remedies in both morbid conditions, much more uniformly than they are in the habit of doing?

One of the most important Chapters of M. Lugol's work is that entitled, "Of the acquired health of parents who engender Scrofulous children." As the epithet *acquired* is here used in opposition to that of *hereditary*, the drift of his remarks is to point out those extraneous influences or conditions which are liable to occasion the development of Scrofula in a constitution that was originally free from any taint of this Cachexy.

I. The first of these that we shall mention, is the existence of Syphilitic Disease in either of the parents. This is unquestionably one of the most frequent occasional causes of Scrofula. Many scrofulous diseases have, it is well known, a striking resemblance with syphilitic maladies. The Ulcers, Ophthalmiæ, Exostoses, Caries, White-swellings, &c. induced by Scrofula are often most perplexingly like to the same diseases arising from a syphilitic cause. This community of the external signs of the two morbid states is, in some degree at least, attributable to the circumstance that scrofulous diseases have often a syphilitic origin. *Astruc* carried his opinion on this subject so far as to maintain that, whenever Scrofula cannot be traced to hereditary descent, it is attributable to a syphilitic taint; and not a few of the most distinguished physicians of the last century allowed themselves to be so far misled by this assertion, as to adopt the same treatment, viz. the use of preparations of mercury, in both diseases. Such a mistake is of truly serious importance; as it is now very generally admitted by all sound practitioners, that the administration of this mineral for any length of time, is most injurious in genuine Scrofula; whereas it is almost indispensable for the proper cure of Syphilis.

II. The second occasional cause of Congenital Scrofula that we shall specify is the abuse of venereal pleasures on the part of parents. "If the seminal fluid," says our author, "be secreted immediately before its emission; if it only passes through the reservoirs, in which it ought to remain for some time; and if it be expelled before its integrant molecules be blended in an intimate and homogeneous manner, it is still imperfect and immature, deficient in prolific qualities, incapable of engendering a healthy and robust offspring: the embryo is stamped with a primal weakness, in consequence of which all the phases of its development are retarded and rendered incomplete." This state of things is of frequent occurrence, as we might anticipate, among the luxurious classes of society; and is accordingly a frequent cause of scrofulous disease among their children.

III. Very early marriages of either sex, before the system has acquired its full tonicity and power, are apt to be followed by similar consequences. A man should not marry before the twenty-fifth year of his age; all marriages, contracted before this period of life, must be regarded as precocious, and consequently liable to be followed by a feeble progeny. The lower animals do not seek for copulation, before their full growth is attained; and even in the case of the vegetable world, we find that the fruit of a tree's first growth is always scanty in number and of indifferent quality: it is only after it has struck its roots more deeply into the earth, and thereby acquired its mature strength and vigour, that its produce becomes full-flavoured and abundant. On this point, *M. Lugol* makes a remark that is somewhat amusing from its pure nationality. He tells us that, in consequence of too early marriages, and the not unfrequent dissipation of the fathers, "the aristocratic families of most countries are desolated by scrofula, and almost extinguished by the hereditary progress of this disease. This is the case more especially in Spain, Italy, England, and Russia; and very probably among the privileged classes of society in all nations." The omission of the French, and the insertion of the English, noblesse in this category, are more creditable to our author's patriotic feelings than to his knowledge of the truth or to his candour.

The opposite extremes of the social community are, in the point of view we are now considering, very nearly alike. What with premature marriages on the one hand, and an insufficient supply of food and clothing on the other, coupled too frequently at the same time with inordinate bodily fatigue —can we be surprised at the amount of scrofulous disease that is annually engendered among the working classes? The following picture of the condition of the French artizan and agricultural labourer will be read with more than ordinary interest at the present time, when every thing appertaining to the state of the poor in our own country is engaging so much attention :—

"Most people are much mistaken in their ideas as to the constitution and mode of existence of our agricultural labourers: they are far from being so contented or so robust as is generally imagined. It is all very fine to talk of their vigorous health and comfortable condition; but the experience of the last ten years, that I have lived in the country (for at least six out of every twelve months), has quite satisfied me that our country population, on the whole, is not of a sound growth, (d'une belle venue), and that most of the inhabitants *age* prematurely. Their food, clothing, dwellings, and general condition are altogether very unsatisfactory. While the amount of food which they have is too little by one-third at least, their amount of labour is certainly a third beyond the average strength of a human being. This disproportion between the amount of labour and the supply of food is, we regret to say, common alike to our country population and the artizans in our manufacturing cities. The children, also, are set far too soon to work, and are kept too long at it. Besides being imperfectly fed, their moral education is quite neglected. Under the influence of this brutalizing system of bodily privation and fatigue, the evil effects of which are too often aggravated by vicious indulgences, it is utterly impossible that these children should grow up to a healthy and robust maturity."

Compare these remarks of *M. Lugol* with the following passage from Dr. Smith's work :—

"In many country districts, where, as regards fresh air and locality, there are all the natural *agremens* of health, scrofula is nevertheless excessively prevalent, and without doubt chiefly on account of an improper or deficient supply of food. There are but too many parishes in England in which the poor population seldom have in their houses any other animal food than salted pork or bacon, and are thus living on a description of food most likely in our climate to debase their physical condition, and produce scrofula, and other diseases which take their rise in the scrofulous constitution.

"Scrofula is fearfully prevalent among the inmates of the present union workhouses. I look upon the New Poor-law as little better than a vast scheme for scrofulising the whole pauper population of Great Britain. Under the poor-law rule, able-bodied paupers, as they are somewhat wrongly termed, are forced to work hard, and the amount of their food is so inadequate to supply the wants of the system, and maintain the expenditure of muscular power, that the constitution inevitably becomes bankrupt in health and strength. These are the beings, who are becoming the parents of a pauper race of children, who in their turn can scarcely hope to reach the original standard of English organization. The evils of the poor-law dietaries are thus reflected back upon the face of society, and they will in the end work out their own punishment by giving us an inferior race of peasants, soldiers, and artisans. This is an aspect which the matter must, if it continues, ultimately assume; and it seems very questionable, whether it is not a pocket wisdom, and a national foolishness, to curtail the rates by poor dietaries at such a risk of physical degeneration on a scale so immense." *

---

* Dr. Smith asserts that it is well known that scrofula has long prevailed among the boys of Christ's Hospital. He attributes the evil to the faulty character of the diet that is adopted in that institution; the food, he says, not being sufficiently varied, and the use of fresh vegetable food being almost entirely excluded. We have good reason to believe that these statements are

IV. When one or both parents are considerably advanced in life, before they have children, it is not unfrequent that the offspring manifests very decided signs of scrofulous cachexy. It is a well-known and popular remark, that old men's children are generally puny and ailing; there is much truth in the observation.

The decrease in the energy of the reproductive powers in the male may be dated from the forty-fifth year of life, or thereabouts. Not very decided at first, it becomes more and more so as years advance; just as the shortening of the days is the more conspicuous, in proportion as we recede from the summer solstice. The first child or two of a quinquagenarian may perhaps be vigorous and robust; but, if the number of his family increases, the younger children are usually much more feeble. "From the fifty-second year," says our admonitory author, "a man is no longer in the physiological condition that is necessary to procreate a being, whose first *besoin* is to grow and enlarge, and whose most rapid growth should occur immediately after conception. At this period of life, a prudent man will the rather abstain from marriage, seeing that, if he has children, he cannot reasonably hope to see them attain maturity, and he must therefore leave them at a tender age without a father to direct their steps."

The same remarks are, in M. Lugol's opinion, applicable to the female sex. When a woman approaches the critical age, her fecundity, which must soon cease altogether, is already much enfeebled; and the child or children, which she may happen to bear, are seldom endowed with the germs of a robust and vigorous life. A woman, if she conceives for the first time after her fortieth year, rarely carries the child to the full period; and if it does survive to be reared, in most instances, alas! it early exhibits some of the characters of scropula.

There are facts of another sort, which serve to show that the circumstance of the age of parents may have much to do with the development of this disease in their offspring. If a couple, after having lived together childless for a multitude of years, are at length blessed with an heir, how often does the child come into the world with the stamp of a congenital weakness:—that is too often the beginning of some scrofulous disease to which it falls a victim in the first or second year of its age! If, again, a long interval of time has elapsed between the births of the ultimate and penultimate children of a family, the former is not unfrequently observed to be much more puny and ailing than the rest.

V. An essential condition of a robust progeny is, that the father be a few years older than the mother. "I have seen," says M. Lugol, "many cases of Scrofula in which I have not been able to discover any other cause, save the disproportionate age of the parents, the father being younger than the mother......... This cause is, however, common enough to have enabled me on many occasions to recognise its operation with facility." Should

---

exceedingly inaccurate: they should therefore not have been made without due enquiry. A copy of the dietary now lies before us; but it is unnecessary to specify particulars. We are moreover informed that very few of the boys are affected with any form of scrofulous disease.

there be any hereditary taint existing at the same time, the disparity in question will tend to aggravate the mischief.

Here it may be remarked that the vigour of the offspring seems on the whole to depend more on the health of the father than on that of the mother. Breeders of cattle certainly attach more importance to the qualities of the male than to those of the female parent. Moreover, we not unfrequently see a delicate woman give birth to fine healthy children, if the father be robust: the reverse of this position is of much less frequent occurrence.*

Besides the sexual circumstances which we have now enumerated as bearing upon the question of the origination of Scrofula in a family, it is more than probable that the offspring of persons, who have, at any period of their lives, been affected with Palsy, Epilepsy, Insanity, or Scirrhus, are more than usually apt to exhibit some traces of the cachexy, although there be no hereditary taint in the family.

The obvious inference to be drawn from all the preceding observations is clearly this : that whatever serves to reduce and exhaust the physical energies of the body, more especially those of the reproductive organs, must tend to promote the development of Scrofulous disease in the offspring. If such be a law of Generation, we cannot wonder at the enormous amount of the evil throughout every nation of Europe, and above all in every large manufacturing city. The luxury and dissipation of the nobility and wealthy gentry; the ceaseless application to business, with its numerous carking toils and cares, of the professional man and the trader; and the exhausting labour and stinted food of the lower classes—all work to the same pernicious end, viz. the increase of scrofulous disease among the rising generation.

The condition of the women among the lower classes is even more unfavourable than that of the men. Their never-ceasing toils and anxieties leave them scarcely " aucun loisir pour la vie de reproduction." Take, for example, the too frequent case of the wives of petty tradesmen among us (the French). They live from day to day in a small confined and unwholesome shop. The air is rendered still more impure by the vapour of the little *chaufferette*, that is so commonly used to warm them against the chilly damp of their habitation. Can we wonder that these poor creatures generally suffer from a variety of ailments, not a few of which are dependent upon a relaxed state of the generative organs ? How then should we expect that a woman in such a condition as this can be the mother of a healthy offspring ?

* M. Lugol asserts that there has been an immense extension of Scrofulous disease throughout France during the present century, in consequence of the excessive drain and destruction of the most robust part of her male population, by the sanguinary wars of the Republic and the Empire. The population of the country has therefore, in a great measure, been renewed " par les hommes les moins propres à engendrer des enfans sains et vigoureux." Under the Restoration, the government found considerable difficulty in forming a corps of 2,500 picked men out of a levy of 80,000 soldiers; and it was even judged necessary, in order to complete this light contingent, to lower the standard height, which had originally been fixed at one *metre* and 70 *centimetres*.

the thing is utterly impossible. Worn out with the fatigues of the day, she is quite indifferent at night to the endearments of conjugal life; and, if she does conceive, her system is not vigorous enough to give due nourishment to the child within her.

Our author recurs to this subject in a subsequent part of his volume, and draws a woful picture of the state of most scrofulous women after marriage. Instead of becoming thereby more healthy, as they are often very erroneously led to anticipate, the constitutional taint generally becomes more active and exasperated, and a host of new troubles is induced. Very many of them remain sterile; or, if they conceive, suffer abortions and premature deliveries. When they go their full time, they usually suffer much more during labour than other women. Leucorrhœa is moreover distressingly common in scrofulous females. Besides their physical maladies, these poor creatures are often the victims of the most painful disquietudes of mind. Hence the frequency of those *malheurs et chagrins domestiques,* that mar the peace of married life. "Too often," continues M. Lugol, "Marriage is unadvisedly recommended with the view of invigorating the health of young women. This is a sort of flattery, to which a medical man should never yield his concurrence. On the contrary, it is his mission, and his duty to resist the vanity of parents, and to shew them how apt they are to sacrifice to this feeling the future happiness of their children." As a matter of course he gives a case in point, where a young mother of six children—two of whom had already died—gave vent to the most bitter reproaches against *les sommités medicales qui avaient donné à ses parens le conseil de la marier!* How often, he adds, do we hear the remark that health is the first of blessings; and yet how little is the regard that is paid to secure this foremost good! It would actually seem as if less anxiety were felt, in the present day, about the health of our own offspring, than about that of our domestic animals; for certainly more trouble and pains are taken in selecting a stallion, than a *chef de famille!*

The frequency of Abortion in women of a scrofulous constitution has been alluded to more than once in the preceding pages. Our author goes so far as to assert that one-fourth at least of scrofulous children perish within the womb. According to his views, the cause of Spontaneous Abortion may reside in the state of health either of the father or of the mother.

When the seminal fluid of a man is too degenerate and not duly vivifying in its qualities, its elements cannot blend sufficiently intimately with the elements derived from the female system, to possess a common and consentaneous existence and pass through all the phases of fœtal life. Under such circumstances, Abortion will take place, however healthy the mother may be; just in the same manner as deteriorated grains will not germinate healthfully, even in the very richest soil. I knew, says M. Lugol, a scrofulous man, whose puberty was not mature till he was nearly 30 years of age, and who married two or three years afterwards. His wi.e, who appeared to be in excellent sound health, had repeated miscarriages, and never carried a child to the full term. Now what Scrofula did in this case—and it is far from being an uncommon one—may be owing to debauched habits in another, and to advanced years on the part of the husband in a third.

*Cæteris paribus,* the principal cause of Abortion is the degradation of the generative organs in the male: more rarely it resides in an analogons condition of these parts in the female. Such a condition may be present, and yet the woman, if impregnated by a healthy man, may become the mother of a robust offspring; but we rarely, if ever, observe that a healthy woman has very robust children by a man who is of a feeble and scrofulous constitution,

When the hereditary taint of the fœtus is derived from both parents, a miscarriage is of almost inevitable occurrence; and pregnancy therefore becomes a cruel deception. Should the child survive to the full period, it is only born to suffering and an early death.

It is because Abortions have their source in the faulty health of one or of both parents, that they generally occur without any external cause. It is a common, but a very erroneous, opinion that this accident is often owing to slight casualties; such as a slip of the foot, jolting in a carriage, lifting a weight, a sudden fright, the abuse of venereal pleasures, and so forth. If such were the case, few women would escape miscarrying. The truth is, that these things have little or no effect on women of a decidedly healthy and robust constitution. Nay, is it not well known that even the most violent (so-called) abortive medicines seldom succeed in inducing miscarriage? and, alas! many are the cases, wherein the woman has died from the use of such means, but without the expected effect being produced. Indeed, we do not hesitate to assert that criminal attempts to induce miscarriage—except resort be had to mechanical interference—very seldom succeed.

Hitherto even the most esteemed obstetrical writers have paid little or no attention to the influence of internal or constitutional causes in inducing miscarriage. For example, *Desormaux*—certainly one of the best authorities on the subject—does not even so much as allude to them in his various works. This circumstance is, in itself, sufficient to show how little this point of practice is attended to in our schools of medicine, in the present day. The accident is almost always referred to some merely local cause, or another; and yet an abortion, thus induced, is (we use M. Lugol's own words) *un fait isolé et sans valeur dans l'avenir.*

So great and so varied are the evils, social as well as personal, that arise from the diffusion of Scrofula among a nation, that our author seriously suggests the propriety of governments retaining to themselves a power to regulate—under certain restrictions, as a matter of course—the marriages of such of their subjects as are unfortunately affected with the malady. His reasonings on this delicate point of political ethics will be best understood from the following somewhat fanciful observations.

"A man marries *pour posseder une femme et procreer des enfans sainement organisés:* his object is happiness, moral and physical. But this two-fold object is frustrated, if either parent imparts the germ of hereditary disease. The only disqualification for marriage, according to our Civil Code, is insanity; and this merely because there is, then, the want of free assent in one of the two parties. The State has unfortunately not reserved to itself any power to enquire into the condition of those who marry. I admit that many difficulties stand in the way of any direct interference of the government in this respect; but surely there is no good reason why society should be left unprotected against the propagation of hereditary diseases."

M. Lugol even regards with a tolerant eye the practice of the old Spartans, who, it is well known, put their puny infants to death; but he afterwards guards himself from misconstruction, by saying that it would be much better to interdict marriage, where there is reason to believe that the offspring will inherit a tainted constitution. "It will at once," continues he, "be objected that we have no right to interfere with the personal enjoyments of any one. But such language is only flattering for a moment to vanity, at the expense of health and comfort. How often do we see discord and disunion in a family, caused entirely by the circumstance we are now alluding to! I have myself known the existence of a simple Otorrhœa embroil the peace of a newly-married couple; I have known Insanity and Suicide induced by the dislike thereby occasioned; and too often have I seen young women, thus situated, take refuge in consolations, which have afterwards plunged them, during the rest of their lives, into unavailing remorse!"

After narrating a case in the way of illustration, he exclaims: "What a deplorable existence for a woman! either to have constantly-recurring abortions, or to give birth to children that are inevitably sickly and feeble from the first moment of their existence." His anticipations of the blessings, that would flow from a code of legislation *empreinte de l'esprit physiologique*, may be gathered from the following prediction. "When this (the interdiction of marriage among certain parties) shall be inscribed in our Codes, and when all the world shall be born under the same law, society will soon be peopled with men of a better complexion, and the number of hereditary diseases will gradually become less and less; so that, in the course of three or four generations, they will be of rare occurrence. Moreover, there will be fewer persons who are blind, dumb, deaf, or rickety; there will be fewer orphans, incurable invalids, and infirm old men; the population of our *hospices* will rapidly decrease; and there will be a larger number of healthy robust citizens for the agricultural and manufacturing interests."

How far the strong arm of the law can ever be made available to the interdiction of marriage, in a country that professes to have freedom of thought and action, we must leave to M. Lugol's countrymen to determine. Any attempt to do so would be regarded, we are sure, as at once impious and tyrannical in the extreme, in most nations. Some persons however are, we are assured by our zealous author, very docile and accessible to reason on this point of duty, as to marrying whom and when they please, and will cheerfully be guided by the instructions of their medical attendant. As a matter of course, he gives us a case in proof. "A man, thirty years of age, and who had suffered much from scrofula at a former period of his life, consulted me before he took the serious step of marriage. In giving him permission, if I may say so, I strongly advised him to spend nine months at least of the year in the country; to be a great deal out in the open air; and every now and then to travel about for change of air; to live on a nourishing and generous diet; to avoid all sorts of excesses; to cultivate the sweet affections of home; and altogether to lead a patriarchal life. By following this regimen, he will doubtless enjoy himself as far as is practicable, and *il travaillera sur lui-meme à la regeneration de sa race*."

Dr. Smith has some very sensible remarks on the Marriages of Scrofulous Persons. Their pith is contained in the following extract:

"To sum up the whole matter: those who possess the signs of the strumous temperament, or have reason to suspect their strumous descent, should beyond all things avoid a matrimonial alliance with a blood relation, or with another party of strumous habit; and they should, with almost equal care, avoid a union with another of the same temperament as themselves, even if there be no signs of the strumous diathesis engrafted on the temperament which resembles their own. The union of opposite, or at all events of different, temperaments, is of all means the most likely to remove the signs of struma in the offspring." 155.

As to the commonly-received opinion, that hereditary *Scrofula sometimes passes over one generation to appear in the next*—as seems to be not unfrequently the case with such diseases as Gout and Asthma—M. Lugol strongly protests against its truth. "This doctrine is altogether gratuitous. A man, who is born of scrofulous parents and has children that are so, is scrofulous himself. The proof of this lies in the very state of his own progeny; otherwise we must suppose that a man may communicate to another what he has not got himself; in other words, that there may be an effect without a cause. * * * * * The facts, on which this opinion has been supposed to rest, are quite fallacious. They are similar in all respects to those which we have alluded to before, on the subject of Scrofula being transmitted by parents who themselves seemed to be quite cured of the disease, or who were supposed not to be at all scrofulous, although they had brothers and sisters who were decidedly so."

In examining the question as to the escape of an intermediate generation from the constitutional taint, many circumstances, which are too often overlooked, require to be taken into account. Not a few persons are unable to tell us, with any degree of certainty, whether they were sickly in early childhood or not; and others will do all in their power to conceal the existence, or even the slightest suspicion, of Scrofula in any member of their family, and to divert the attention of the medical man from pursuing the unwelcome subject. It requires, therefore, no ordinary tact and perseverance to arrive at the truth in many instances. The discriminating eye of an experienced physician will, however, often detect the existence—although latent—of the inborn evil, by some trifling peculiarity of bodily conformation which would quite escape the ordinary observer. For example, the joints may be disproportionately large, compared with the size of their shafts, or with the plumpness of the soft tissues; or one or two of the cervical glands may be a little enlarged; or there may be a tendency to weakness of the eyes, or to slight discharge from the nose, or to leucorrhœa, &c. The existence of any one of these conditions is sufficient to declare the existence of a strumous habit, however healthy the individual may *seem*, and assert herself, to be.

Dr. Smith, we may remark, differs entirely from M. Lugol in reference to the question we are now considering. He maintains that, "in a highly scrofulous family, the scourge may pass over one generation without affecting it, but only to revisit the next." He admits, indeed, that this *Atavism*, so to speak, of the disease seems to border on the marvellous; but he fully believes that its truth is completely established, and appeals to his own experience in confirmation of it.

"More frequently," he goes on to say, "it occurs in scrofulous families, that there are some affected in every generation, while others remain pure. The disease, or the tendency to disease, often descends indirectly and irregularly, occurring in uncles and aunts in one generation, and nephews and nieces in the succeeding. I should observe that Sir James Clark believes that the occurrence of scrofula in alternate generations will admit of explanation, without supposing that the disease sleeps in one generation to re-appear in the succeeding. I believe this eminent physician considers these cases are explainable by the actual eradication of the disease in the generation in which it is absent, and its re-development when it appears in the following generation, by the agency of new causes. I submit, however, that there are sufficient reasons for the rejection of this latter doctrine. Such a kind of pathological atavism, or irregular development of disease, has always been so frequent, and so much a matter of common observation, as to be very generally believed. And further, we see the same thing happen with regard to physical organization. It frequently occurs that a child resembles one of its grandparents or an uncle or aunt, in growth, stature, temperament, and mental qualities, while it bears little likeness to its immediate progenitors." 13.

The weight of evidence and force of reasoning seem to us to be decidedly opposed to the opinions of the English author, and in favour of those so strenuously advocated by M. Lugol. We cannot bring a clean thing out of an unclean; and how can the tree, that is corrupt, bring forth good fruit? So it is, most probably, in reference to the transmission of Scrofula—which, be it always remembered, is not so much a disease as an unhealthy constitution or diathesis of the body. We are inclined, therefore, to agree with M. Lugol, that no thoroughly robust and healthy person is ever born of scrofulous parents: *fortes creantur fortibus*. As we have already said, the tendency to the development of the disease in the offspring is greater when the hereditary transmission is derived from the father, than when it is from the mother.

### *Treatment of Scrofulous Diseases.*

It is certainly much rather by following out a judicious hygienic regimen than by the use of any direct medicinal agents, that we can hope to effect a salutary change in the constitution of scrofulous patients. M. Lugol does little more than merely allude, here and there, to the therapeutic part of his subject: his present work does not profess to embrace more than the *etiology* of the disease in question. He takes occasion, however, in more than one passage, to reprobate with marked condemnation the common use of antiphlogistic and other lowering remedies in the maladies of scrofulous children, and the too-prevalent custom of localising, in practice as well as in theory, the morbid action, without paying due attention to the general condition or diathesis of the system. He strongly enjoins free exposure to light and air in the treatment of most forms of the disease. "I have had frequent opportunities," says he, "of observing the bad effects of treating scrofulous Ophthalmia in dark chambers. It is not an uncommon remark with such patients, that they see better on those days when they are taken to the surgeon for advice." He then proceeds to point out the great importance of regular exercise, in the treatment even of White Swelling of the joints. "In my third memoir on the employment of Iodine, published in 1831, I dwelt at some length on the value of this mode of cure—viz. regular bodily exercise, (the patient walking, as a matter of course, on crutches,) along with the use, external and internal, of ioduretted preparations. Since that period, I have uniformly acted upon this principle; and the great success, that has attended my hospital practice, has been publicly testified by Professor Magendie and many others. .......... Why is my practice not more generally followed? Simply because medical men persist in regarding this, and many other forms of scrofulous disease, as of a local nature, and not as the mere expressions of a constitutional Cachexy."

We must now turn to Dr. Smith, who enters much more largely into the subject of the treatment of Scrofula. We shall select a few mems, that may possibly amuse, as well as instruct, the reader.

He recommends the Solution of Gastric-juice or *Pepsin*—as it has of late years been called—not only as a stomachic remedy, but also as a tonic of great efficacy in many cases. He thinks it likely to come into general use: we much doubt it.

"I have," says he, "been in the habit of giving the solution of pepsin about a quarter of an hour after every solid meal, giving a larger quantity after dinner than at any other time. There can be no doubt that it gives the stomach an additional digestive power; and besides increasing the general strength, I have seen it remove the gastrodynia, flatulence, and heat at the epigastrium, which is so common during digestion in delicate persons." 77.

With respect to *iodine*, Dr. Smith is adverse to the large doses in which it and its preparations are so often administered. He prefers giving them in minute doses, largely diluted, and upon an empty stomach.

"A drachm of the iodide of potassium, dissolved in an ounce of distilled water, and this solution given in doses of twelve or sixteen minims three times a day, in a tumbler of water, will bring the system under the effects of iodine in a few days. The peculiar determination to the mucous membrane of the nose, eyes, and fauces will be established, the taste of iodine becomes perceptible in the mouth, and the odour of it in the increased mucous secretion. As another instance of the effects of still smaller doses of iodine in the prevention of disease, I may refer to a statement of Boussingault respecting the comparative frequency of goître among the inhabitants of different parts of the Cordilleras in South America. This chemist states that in certain districts, in which the population use salt impregnated with a very small quantity of iodine, the health of the inhabitants is good; while in others, where salt is used entirely destitute of iodine, goîtrous diseases abound. Dr. John Davy, in referring to the above, says:—'It is not improbable that the apparent increase of scrofulous and consumptive disease in recent times may be connected with the over refinement of salt; that is, carried so far as to deprive it of its iodine principle, which seems intended by provident nature as a corrective of certain injurious causes productive of a terrible class of diseases.'" 91.

But far more efficacious than Iodine, or any other medicinal or hygienic agent, is—can our readers guess?—*the royal touch*.

"It is my intention," says our credulous author, "on the present occasion, to avow a firm belief in its power and efficacy. I cannot agree that a practice followed implicitly for the space of seven centuries in this country alone, besides its long performance in France, and which lived through our golden age of letters, could have been purely fictitious, and not have been demonstrated to be such beyond the power of appeal. Those who saw most of its operation were the firmest of its supporters, and it frequently obtained the testimony of medical practitioners. Thus, Wiseman, serjeant-surgeon to Charles the First, a good authority in scrofula, as he himself practised in this disease, declared that the king cured more scrofulous patients than all the chirurgeons of London put together." 164.

Without recommending, in so many words, the re-adoption of the royal remedy, Dr. *Smith* wishes us, nevertheless, to believe that "no other mental remedy of equal efficacy was ever devised or practised; and certainly, as I believe, nothing of the kind can be resorted to in the present day which approaches it in power." What says he of the wonderful cures that have been and still are (so, at least we are told, and that too in print) effected by the mere sight of holy relics, such as pieces of the true cross, bones of saints, the blood of our Saviour, and so forth? Are *they* not quite as authentic as any thing that has ever been recorded of his favourite mental remedy for Scrofula? It was only the other day that we read (in the Athenæum for Oct. 12th) of the marvellous cures that have been performed in the course of the present season, at Treves, by the mere exhibition of the *real* (as a matter of course) Holy tunic to the gaze of the poor ignorant beholders, who flocked in tens and hundreds of thousands to the shrine of the Cathedral of that city. The lame walked, the blind saw, and the dumb spake! Among the rest, the young Countess Jeanne of Munster, who had lost the use of her legs for upwards of three years, no sooner bent before the relic, and touched the sacred cloth, than her limbs were straightened, her figure became once more erect, and she quitted the Cathedral, leaving her crutches behind her in memory of her miraculous cure!! What will Dr. Smith say to this? Might not the display of such a precious piece of sanctity be still more effectual than even the touch of a royal hand? How far the re-institution of the regal prerogative, in the person of our gracious Queen and Governor, might serve to counteract the scrofulizing effects of the much abused new Poor Law, we must leave to Sir Robert Peel, or some other of our learned senators, to determine.

While this article was passing through the press, we received Dr. Ranking's translation of M. Lugol's Treatise. On the whole, it is well and ably executed; and the profession, we believe, will be pleased to have an English version of this somewhat remarkable work. We cannot, however, concur with Dr. Ranking, in his extravagant laudation of his author's researches—"which," he declares to be, "in extent of benefit conferred, and for advancement of rarely useful medical science, unequalled by the labours of any other individual in any other department of medicine." Few medical men will join in such an exaggerated statement, or agree with Dr. R. in many of his therapeutic rules and suggestions—which, as may be anticipated, are all based on the doctrines of the anti-scrofula physician of St. Louis' Hospital. In our opinion, the whole of the appended Essay on the treatment of the disease is vitiated by a most unwise over-estimate of the curative powers of Iodine and its preparations. For example, we are told that "the only system of treatment, which is really deserving of confidence (in scrofulous diseases of the bones and joints), is the internal and external employment of iodine, combined with such mechanical contrivances as shall enable the patient to be continually in the open air;" and Dr. R. assures us that "he has seen more than one patient, whom a

Lumbar Abscess was daily bringing nearer to the grave, gain appetite and flesh, and the discharge cease in less than a month from the time of commencing the medicine." He recommends, also, that scrofulous glandular abscesses should be opened by incision, as soon as fluctuation becomes distinct, and forthwith he injected with a solution of Iodine—a practice that few practical surgeons, we should think, will follow; even though it be followed by M. Lugol himself. In the treatment of Porrigo and the severer forms of Eczema and Impetigo, the internal use of the medicine is deemed almost indispensable; and in strumous Ophthalmia it constitutes, in Dr. Ranking's opinion, our sheet-anchor of hope:—the Quinine, although a most valuable tonic, "having no special effect upon the tubercular constitution, and being therefore unable to prevent the liability to relapse." The Liquor Potassæ is regarded by him as equally defective in this point of view; the preparations of Steel are only incidentally mentioned; and the effects of warm clothing are scarcely so much as alluded to.

The only other topic that we can at present notice is the advice contained in the following passage:

"When, from the known peculiarities of the parents, an infant is suspected to be scrofulous, it should, unless the contamination be clearly confined to the father, be intrusted immediately to a wet-nurse. The mother ought not, in any case, to suckle her child, however well she may be prepared so to do, if she or her family exhibit the scrofulous diathesis." So unqualified a recommendation as this is surely most injudicious; and even were it not so, is it not obvious that it would be nearly impracticable? Where are the wet-nurses to be found for every child of every woman, in whom, or in whose family, there is a scrofulous taint? and what, pray, is to become of the nurses' own poor children deprived of their natural food, and thereby rendered subject to that very disease, which Dr. Ranking seeks, in rather a strange manner, to eradicate? Thank heaven, there is no occasion for the sweeping condemnation of suckling to so large a number of mothers, that is laid down in the paragraph which we have quoted. That scrofulous women should suckle their children *less* than those of a more robust constitution, we readily admit; but that they ought not, in any case, to do so at all, is an injunction that seems to us to be alike injudicious and unrequired.

